# Postpartum quality of life and associated factors: a cross-sectional study

**DOI:** 10.1007/s11136-023-03384-3

**Published:** 2023-04-10

**Authors:** Bushra O. Al Rehaili, Rajaa Al-Raddadi, Nadiyah Karim ALEnezi, Ala H. ALYami

**Affiliations:** 1 Saudi Board of Preventive Medicine, Al Madinah Residency Program of Preventive Medicine, Ministry of Health, Madinah, Kingdom of Saudi Arabia; 2 Department of Community Medicine, Faculty of Medicine, King Abdulaziz University, Jeddah, Saudi Arabia; 3 Saudi Board of Preventive Medicine, Ministry of Health, Madinah, Kingdom of Saudi Arabia; 4 Saudi Board of Preventive Medicine, Ministry of Health, Tabuk, Kingdom of Saudi Arabia

**Keywords:** Quality of life, Postpartum depression, Saudi Arabia, Social support

## Abstract

**Purpose:**

To determine factors associated with postpartum quality of life (QOL).

**Methods:**

An analytic cross-sectional design was used in this study, and data was collected from December 2019 to March 2020. Participants were 252 postpartum women visiting eight governmental primary healthcare centers in Madinah city, Saudi Arabia. Data were collected using a sociodemographic questionnaire, the World Health Organization Quality of Life Assessment-BREF, Multidimensional Scale of Perceived Social Support (MSPSS), and Edinburgh Postnatal Depression Scale. Sleep problems were assessed using an item from the Prime-MD Patient Health Questionnaire.

**Results:**

Maternal age between 26 and 35 years had a significant independent association with the physical health domain of QOL (*p* < .01). Postpartum depression was significantly associated with lower QOL in all dimensions (*p* < .01). In addition, sleep problems were associated with three out of the four QOL domains (*p* < .05). The significant other subscale of the MSPSS was significantly associated with higher QOL scores in all dimensions (*p* < .01); additionally, family and friends subscales of the MSPSS were significantly associated with the social domain of QOL (*p* < .01).

**Conclusions:**

Maternal QOL, during the postpartum period, showed negative associations with age, postpartum depression, and sleep disturbances. Ultimately, social support appeared to be an essential factor in mothers’ ability to cope with the physical and psychological problems experienced during this period.

## Introduction

*Quality of life* (QOL) has been recognized as a substantialoutcome measure for health service evaluation [1]. QOL is defined as individuals’ perception of their position in life in the context of their culture and value systems, which concerns their targets and expectations [2]. In the area of maternal care, decreasing morbidity and mortality rates have prepared the ground for other goals such as enhancing QOL [3]. With regard to QOL during periods of childbirth and childcare, although health professionals are involved in prenatal care, postpartum maternal healthcare is relatively neglected [4].

The *postpartum period* is a time of transition in a mother’s life marked by physical, psychological, and social changes [5] that begins after childbirth and lasts up to 6 months. It is often divided into three distinct phases: initial or acute phase, which lasts 6–12 h postpartum; second or subacute postpartum period, which lasts 2–6 weeks; third or delayed postpartum period, which can last up to 6 months [6]. Recent studies have quantified problems occurring during the postpartum period, such as mental distress, genital infections, physical complaints, and sleep problems [7, 8]. Sociodemographic variables such as maternal age, educational level, and reproductive history have been shown to be significant influencing factors across the postnatal period [9, 10]. Among the other factors associated with QOL, postnatal depression and social support have been reported to be significant [11, 12]. Furthermore, maternal sleep disturbances are of concern because of their association with fatigue and depression, which are strongly associated with QOL [13, 14].

In Saudi Arabia, the prevalence of postpartum depression (PPD) ranges from 14 to 17.8% [15, 16]. While several studies suggest a significant association between QOL and symptoms of PPD [17, 18], postpartum QOL is not well researched in Saudi Arabia. Existing studies have generally focused on physical postpartum complications such as hemorrhage and urinary incontinence [19, 20] PPD [21] and the association of postpartum QOL with a particular type of delivery [5]. Other important factors considering Saudi Arabia’s context and social diversity are not well investigated. Hence, this study aims to assess maternal QOL during the postpartum period and explore its correlates in a sample of Saudi Arabian women.

## Materials and methods

### Study design and setting

This cross-sectional analytical study was conducted from December 2019 to March 2020 at eight governmental primary healthcare centers in Madinah city, Saudi Arabia. The participants were recruited through multi-stage sampling. In the first stage, we did a cluster sample to choose 8 PHCs out of the 54 centers in Madinah city; Two primary healthcare centers were randomly selected from each region (i.e., east, west, north, and south). In the second stage, women who attended well-baby clinics at the selected primary healthcare centers and who had a live birth in the last 6 months were invited to participate in the study.

### Data collection tools

Participants completed a self-report questionnaire covering sociodemographic data and maternal and infant history. Sociodemographic data included age, occupation, educational level, income, and prenatal and postnatal history, such as type of delivery and time since previous delivery. Questions regarding the infant included sex, feeding method, daycare (i.e., home with mother, babysitter, or nursery), and health status. Furthermore, the following existing measures were used: the World Health Organization Quality of Life Assessment-BREF (WHOQOL-BREF), Multidimensional Scale of Perceived Social Support (MSPSS), maternal sleep measurements, and Edinburgh Postnatal Depression Scale (EPDS).

#### World Health Organization Quality of Life Assessment-BREF

The WHOQOL-BREF is a short version of the WHOQOL-100 instrument used for measuring QOL in diverse cultural settings [2]. This tool includes 26 items across four domains—physical health, psychological health, social relationships, and environment—rated using a 5-point Likert scale [2]. Raw subscale scores are converted to a 0–100 scale; higher scores indicate better QOL. The WHOQOL-BREF has been validated in a range of languages, including Arabic (Cronbach’s alpha ≥ 0.7) [22]. Additionally, the questionnaire has been validated among women in the postpartum period [23]. 

#### Multidimensional Scale of perceived social support

The MSPSS is a 12-item self-report tool used for measuring perceived social support from three sources: family, friends, and significant other. Items are scored on a 7-point Likert scale ranging from 1 (*very strongly disagree*) to 7 (*very strongly agree*). The mean total score ranges from 1 to 7; higher scores indicate higher levels of perceived social support [24]. The scale is well-validated, showing strong internal consistency, with an overall Cronbach’s α ranging from 0.84 to 0.92 [25]. The Arabic version has been validated, with an overall Cronbach’s α of 0.87 [26].

#### Maternal sleep measurements

Sleep problems were assessed using an item from the Arabic version of the Prime-MD Patient Health Questionnaire: “Trouble falling or staying asleep, or sleeping too much.” Respondents were asked if they were bothered by the problem over the past 2 weeks. The response options were “not at all,” “several days,” “more than half the days,” and “nearly every day,” with scores ranging from 0 to 3 [27].

#### Edinburgh Postnatal Depression Scale

The EPDS is a self-report tool widely used to screen for PPD [28]. It consists of 10 items and addresses the intensity of depressive symptoms over the previous 7 days. Items are scored on a 4-point Likert scale ranging from 0 (*no symptoms*) to 3 (*marked presence or change*), resulting in a total score of 0–30. The Arabic version of the EPDS has been validated in a sample of Emirati women, with a Cronbach’s α of 0.84. [29] 

### Ethical considerations

Approval for the study protocol and data collection procedure was obtained from the Research Ethical Committee in Madinah’s Ministry of Health (IRB 356). The study's objectives were explained to the participants, and written informed consent was obtained.

### Statistical analysis

Data were analyzed using SPSS version 25 (IBM Corp., Armonk, NY, USA). *p*-values < .05 were considered statistically significant. Descriptive statistics were presented as mean and standard deviation for continuous variables, and as frequency and percentage for categorical variables. Student’s *t*-test and ANOVA were used to compare mean scores across QOL domains. Pearson’s correlation was used to test the correlation between mean scores of the MSPSS, EPDS, and QOL domains. Multiple linear regression was used to identify the variables with independent associations with QOL during the postpartum period.

## Results

### Sample characteristics

The data of all 252 women who were approached to participate in the study were analyzed. The participants had a mean age of 31.3 (± 6.2) years, and most were between 26 and 35 years (56%). Additionally, 58.7% were housewives and 35.7% were working. Regarding health status, 12.7% of the sample reported a chronic condition and 2.4% had mental health problems. In terms of obstetric history, 65.1% had experienced vaginal delivery. Regarding parity, 25.8% of women in the study were primigravida.Other characteristics are presented in Table 1. The mean total scores for the WHOQOL-BREF and the MSPSS were 95.2 (±16) and 5.3 (±1.2), respectively. The individual domain scores are shown in Table 2. Considering PPD, the mean EPDS score was 9.9 (±5.3). Regarding sleep problems over the past 2 weeks, participants’ responses indicated 46.0, 22.2, 21.4, and 10.3% for “several days,” “nearly every day,” “more than half the days,” and “no problems,” respectively.

**Table 1:** Distribution of socioeconomic and obstetric characteristics among participants (*N* = 252)

Variable	*n* (%)
Age (years)	
≤25	48 (19)
26–35	141 (56)
≥ 36	36 (25)
Educational Level	
Elementary	14 (5.6)
Secondary	56 (22.2)
University	182 (72.2)
Occupation	
Working	90 (35.7)
Housewife	148 (58.7)
Student	14 (5.6)
Income (USD)	
< 1330	93 (36.9)
1330–2660	91 (36.1)
˃2660	68 (27)
Chronic diseases	
Yes	32 (12.7)
No	220 (87.3)
Mental health problems	
Yes	6 (2.4)
No	246 (97.6)
Delivery mode	
Vaginal	164 (65.1)
Cesarean section	88 (34.9)
Parity	
Primigravida	65 (25.8)
Multigravida	187 (74.2)
Time since previous pregnancy (years)^a^	
Up to 2	41 (16.3)
≥2–4	63 (25.0)
≥4	74 (29.4)
Missing	9 (3.6)
Number of children	
1	65 (25.8)
2–4	145 (57.5)
≥ 5	42 (16.7)
Infant’s sex	
Boy	128 (50.8)
Girl	124 (49.2)
Infant’s age (months)	
1–3	100 (39.7)
3–6	152 (60.3)
Infant health problems	
Yes	14 (5.6)
No	238 (94.4)
Feeding	
Breastfeeding	53 (21)
Formula feeding	87 (34.5)
Mixed feeding	112 (44.4)
Daytime care (nursery)	
Yes	70 (27.8)
No	182 (72.2)

^a^Time since previous pregnancy (years) measured for Multigravida, *N* = 187

**Table 2:** Distribution of mean score of WHOQOL-BREF, MSPSS, and other variables

Variable	Mean (SD)
WHOQOL-BREF	
Total score	95.2 (16)
Physical health	14.5 (2.6)
Psychological health	14.4 (2.8)
Social relationships	14.5 (3.7)
Environment	14.7 (2.9)
EPDS	9.9 (5.3)
MSPSS	
Total score	5.3 (1.2)
Significant other	5.9 (1.4)
Family	5.7 (1.3)
Friends	4.4 (1.7)
Sleep problems	
Total score	1.5 (0.9)
Not at all	26 (10.3)^a^
Several days	116 (46.0)^a^
More than half the days	54 (21.4)^a^
Nearly every day	56 (22.2)^a^

^a^Frequency (%)

WHOQOL-BREF: World Health Organization Quality of Life Assessment-BREF, higher scores indicate better QOL; MSPSS: Multidimensional Scale of Perceived Social Support, higher scores indicate higher levels of perceived social support; EPDS: Edinburgh Postnatal Depression Scale ; higher scores indicate depress

### Factors associated with postpartum QOL

The highest mean QOL scores were among mothers aged under 26 years. The physical health domain score was statistically significantly different by maternal age group (*p* = .03). Mothers of infants aged between 1 and 3 months had a worse score on physical health domain 62.8 (± 16.3; *p* = .02). Pearson’s correlation was used to assess the relationship between scores of the EPDS, sleep, MSPSS, and QOL domains (Table 3). There was a statistically significant correlation between the mean scores of various domains of QOL and EPDS score (*p* < .001). In addition, sleep problems were statistically significantly correlated with the mean total score of QOL and the mean scores of the domains of physical health, psychological health, and environment (<.01). Moreover, there was a statistically significant positive correlation between MSPSS mean total score and QOL domains (*p* < .001). The significant other and family subscale scores were also significantly correlated with QOL domains (*p* < .001). The friends subscale showed a significant correlation with the psychological health and environment domains of QOL (*p *< .01), as well as with the social relationships domain (*p* < .001).

**Table 3:** Pearson’s correlation of scores for the EPDS, sleep, MSPSS, and WHOQOL-BREF domains among participants

	Total QOL score	Physical health	Psychological health	Social relationships	Environment
EPDS	−.554**	−.506**	−.515**	−.442**	−.458**
Sleep problems score	−.301**	−.425**	−.228**	−.120	−.246**
MSPSS					
Total score	.458**	.271**	.385**	.505**	.453**
Significant other	.543**	.367**	.446**	.510**	.560**
Family	.387**	.272**	.350**	.301**	.389**
Friends	.231**	.065	.184*	.429**	.204*

EPDS: Edinburgh Postnatal Depression Scale, MSPSS: Multidimensional Scale of Perceived Social Support, WHOQOL-BREF: World Health Organization Quality of Life Assessment-BREF

*Statistically significant at *p* value < .01, **p value < .001

### Correlates of QOL during the postpartum period

The multiple regression models are presented in Table 4. The results of the final models revealed that the identified correlates explained 34–43% of the variance in the various QOL domains. PPD was significantly associated with lower QOL in all dimensions (*p* < .01). In addition, sleep problems were associated with three of the four QOL domains (*p* < .05). Maternal age between 26 and 35 years displayed a significant independent association with the physical health domain (*p* < .01). The significant other subscale of the MSPSS was significantly associated with higher QOL scores in all dimensions (*p* < .01). Additionally, the family and friend subscales of the MSPSS were significantly associated with the social relationships domain of QOL (*p* < .01).

**Table 4:** Multiple linear regression analysis results for domains of the WHOQOL-BREF

	*B*	95% confidence interval		SE B	*Β*	Sig.	*R*^2^	$\Delta$*R*^2^
		LL	UL					
Physical health								
Constant	75.25	65.05	85.44	5.17		.000	**.45**	.43*
Age (reference: ≤ 25 years) 26–35	−5.72	−9.87	−1.56	2.10	−.172	.007		
≥ 36	−2.005	−6.75	2.74	2.41	−.053	.407		
EPDS	−.962	−1.29	−.634	.167	−.311	.000		
Sleep problems (scores)	−3.186	−4.05	−2.31	.44	−.364	.000		
Significant other, MSPSS	2.444	.902	3.98	.78	.209	.002		
Family, MSPSS	.666	−.925	2.25	.808	.054	.411		
Friends, MSPSS	−.806	−1.88	.271	.547	−.082	.142		
Psychological health								
Constant	57.76	46.78	68.73	5.57		.000	**.35**	.34*
EPDS	−1.18	−1.55	−.812	.190	−.362	.000		
Sleep problems (scores)	−1.15	−2.14	−.164	.503	−.124	.023		
Significant other, MSPSS	3.28	1.54	5.02	.885	.265	.000		
Family, MSPSS	.864	−.945	2.67	.919	.066	.348		
Friends, MSPSS	−.281	−1.49	.933	.616	−.027	.649		
Social relationships								
Constant	42.11	28.25	55.96	7.03		.000	**.40**	.39*
EPDS	−1.34	−1.80	−.884	.234	−.306	.000		
Significant other, MSPSS	6.421	4.178	8.66	1.13	.387	.000		
Family, MSPSS	−3.202	−5.53	−.870	1.18	−.183	.007		
Friends, MSPSS	3.99	2.431	5.55	.792	.288	.000		
Environment								
Constant	46.28	35.44	57.11	5.50		.000	**.41**	.40*
EPDS	−.821	−1.19	−.452	.187	−.243	.000		
Sleep problems (scores)	−1.401	−2.37	−.424	.496	−.147	.005		
Significant other, MSPSS	5.86	4.145	7.58	.873	.459	.000		
Family, MSPSS	.345	−1.44	2.13	.907	.026	.704		
Friends, MSPSS	−.591	−1.79	.608	.609	−.055	.332		

EPDS: Edinburgh Postnatal Depression Scale, MSPSS: Multidimensional Scale of Perceived Social Support, WHOQOL-BREF: World Health Organization Quality of Life Assessment-BREF, LL: lower limit, UL: upper limit

*Statistically significant at *p* < .01

## Discussion

Numerous studies in Saudi Arabia have evaluated the physical and mental challenges that arise during the postpartum period. However, to the best of our knowledge, this is the first to assess QOL and its correlates, including PPD and social support, in this phase. Our findings showed maternal age between 26 and 35 years was significantly negatively associated with the physical health domain of QOL compared to younger mothers. There were also significant correlations between QOL domains and symptoms of PPD, sleep problems, and the significant other subscale of social support. Further, the family and friend subscales were ass ociated with the social domain of QOL.

In our study, mothers aged 26–35 years had lower scores on the physical health domain of QOL compared to mothers younger than 25 years. Similarly, a recent study observed that women younger than 30 years had better QOL on three subscales of the 36-item Short Form Survey: social functioning, bodily pain, and vitality (energy and fatigue) [30]. This is probably explained by the fact that younger mothers are mostly primiparas and receive more attention and support from their partners and families than their older counterparts. Similarly, a Turkish study found that spousal support and QOL decrease with maternal age [12].

The present study revealed that depression is an independent risk factor associated with poor scores in all the domains of QOL. Several studies have reported the negative impact of depression on QOL during the postpartum period [31]. A study conducted in Kuwait showed that health-related QOL and the physical and mental health domains of QOL were associated with PPD [32]. Zubaran et al. investigated QOL and depressive symptoms among Brazilian women and reported a mean EPDS score of 8.35 (±5.6), which was significantly correlated with all domains of the WHOQOL-BREF [33]. Moreover, PPD negatively affects the mother, infant, and family relationships [34]. Evidence shows that the risk of PPD increases during the first 3 weeks after delivery and continues for almost 2 years [35]. However, in an Iranian study, the mean depression score decreased significantly from 6 to 8 to 14 weeks after delivery [36]. Furthermore, many studies have shown the protective role of social support in PPD [37]. Additionally, 12.7% of our sample had chronic medical conditions that could affect their experience of postpartum depression. Studies have found that pregnant women with chronic medical conditions were more likely to develop mental illnesses around childbirth, including postpartum depression [38].

Social support is a crucial component of the physical and emotional well-being of mothers following childbirth [39]. The current study showed that mothers received high levels of social support; the mean total MSPSS score was 5.3 (±1.2). A high level of social support for new mothers is a Saudi cultural norm; women receive a lot of help in the transitional time after delivery and the mother often spends the puerperium period, particularly the first forty days, at her mother's house to receive the necessary support until she recovers, which is necessary if she is new mother.Increased social support was found to be correlated with fewer physical role limitations and higher levels of general and mental health among postpartum women [40]. A recent Japanese study that explored factors associated with QOL among women rearing a 4- or 18-month-old child showed that emotional support from families was closely correlated with QOL [41]. Another study examining the impact of social support on QOL (measured by the WHOQOL-BREF) following delivery reported that women with low partner or family support had lower scores across all QOL domains compared to well-supported women [42]. Correspondingly, our findings reveal that social support, in general, is associated with better QOL scores in all domains. In addition, help from a significant other was independently associated with QOL. Several studies have identified the positive influence of spousal support on QOL in women after childbirth [12, 41]. A study among postpartum Saudi women observed that 84% of participants had a good perception of partner support and were able to consider their extended family members, parents, and relatives as their primary help providers. [43]

This study identified sleep problems as risk factors ofthree of the QOL domains (i.e., physical health, psychological health, environment) was identified in this study, similar to previous studies. For example, a study found that for mothers of 4-month-old infants, getting enough sleep enhanced QOL across all domains except social relationships [41]. Moreover, women in the postpartum period are more likely to experience interrupted and lower-quality sleep. Based on the Pittsburgh Sleep Quality Index total score, a study among postnatal women found that 59% reported poor sleep quality, and 16% reported medium or high sleep disturbance [44]. In our study, 22% of mothers experienced sleeping difficulties nearly every day. Postpartum sleep quality is affected by fatigue, frequency of nighttime awakening, mood disturbance, and childcare demands [45, 46]. Moreover, poor sleep quality was linked to greater depressive and anxiety symptoms in women who had 6-month-old infants [47].

### Limitations

There are some limitations to our study. First, it is cross-sectional in nature; thus, we were unable to make causal inferences regarding the relationships among study variables. Second, a relatively small sample was used. Additional research using a longitudinal design is required to investigate QOL changes during postpartum period.

## Conclusion

Postpartum QOL negatively correlated with maternal age, PPD, and sleep disturbances. Ultimately, positive social support is essential for the mother to cope with the physical and psychological problems experienced during the postpartum period.

## Recommendation

Our findings suggest the importance of comprehensive care for mothers in the postpartum period. Healthcare providers need to be aware of the importance of QOL and how numerous variables influence it. PPD screening and assessment of sleep quality should be considered during postnatal visits. In addition, social support plays a positive role in maternal QOL. Healthcare providers need to be conscious of mothers’ social circumstances during antenatal follow-up and encourage them to seek help if required.
